# 9-ING-41, a Small Molecule Inhibitor of GSK-3β, Potentiates the Effects of Chemotherapy on Colorectal Cancer Cells

**DOI:** 10.3389/fphar.2021.777114

**Published:** 2021-12-09

**Authors:** Andrey Poloznikov, Sergey Nikulin, Larisa Bolotina, Andrei Kachmazov, Maria Raigorodskaya, Anna Kudryavtseva, Ildar Bakhtogarimov, Sergey Rodin, Irina Gaisina, Maxim Topchiy, Andrey Asachenko, Victor Novosad, Alexander Tonevitsky, Boris Alekseev

**Affiliations:** ^1^ Faculty of Biology and Biotechnologies, Higher School of Economics, Moscow, Russia; ^2^ P. Hertsen Moscow Oncology Research Institute–Branch of the National Medical Research Radiological Centre of the Ministry of Health of Russian Federation, Moscow, Russia; ^3^ School of Biomedicine, Far Eastern Federal University, Vladivostok, Russia; ^4^ Scientific Research Centre Bioclinicum, Moscow, Russia; ^5^ Engelhardt Institute of Molecular Biology, Russian Academy of Sciences, Moscow, Russia; ^6^ Department of Surgical Sciences, Uppsala University, Uppsala, Sweden; ^7^ Department of Pharmaceutical Sciences, College of Pharmacy, University of Illinois, Chicago, IL, United States; ^8^ A. V. Topchiev Institute of Petrochemical Synthesis, Russian Academy of Sciences, Moscow, Russia; ^9^ Laboratory of Microfluidic Technologies for Biomedicine, Shemyakin-Ovchinnikov Institute of Bioorganic Chemistry RAS, Moscow, Russia

**Keywords:** colorectal cancer, KRAS, tumor organoids, GSK-3, 9-ING-41, cell cycle

## Abstract

Colorectal cancer (CRC) is one of the most common and lethal types of cancer. Although researchers have made significant efforts to study the mechanisms underlying CRC drug resistance, our knowledge of this disease is still limited, and novel therapies are in high demand. It is urgent to find new targeted therapy considering limited chemotherapy options. *KRAS* mutations are the most frequent molecular alterations in CRC. However, there are no approved K-Ras targeted therapies for these tumors yet. GSK-3β is demonstrated to be a critically important kinase for the survival and proliferation of K-Ras–dependent pancreatic cancer cells. In this study, we tested combinations of standard-of-care therapy and 9-ING-41, a small molecule inhibitor of GSK-3β, in CRC cell lines and patient-derived tumor organoid models of CRC. We demonstrate that 9-ING-41 inhibits the growth of CRC cells *via* a distinct from chemotherapy mechanism of action. Although molecular biomarkers of 9-ING-41 efficacy are yet to be identified, the addition of 9-ING-41 to the standard-of-care drugs 5-FU and oxaliplatin could significantly enhance growth inhibition in certain CRC cells. The results of the transcriptomic analysis support our findings of cell cycle arrest and DNA repair deficiency in 9-ING-41–treated CRC cells. Notably, we find substantial similarity in the changes of the transcriptomic profile after inhibition of GSK-3β and suppression of STK33, another critically important kinase for K-Ras–dependent cells, which could be an interesting point for future research. Overall, the results of this study provide a rationale for the further investigation of GSK-3 inhibitors in combination with standard-of-care treatment of CRC.

## Introduction

Colorectal cancer (CRC) is one of the most common cancers worldwide. More than 1.9 million new CRC cases and 935,000 deaths were reported in 2020. Overall, CRC ranks third in terms of incidence and second in terms of mortality (Sung et al., 2021). 5-FU–based chemotherapeutic regimens, such as CAPEOX, FOLFOX, and FOLFIRI, are recognized as a standard of care (SoC) treatment for the metastatic CRC (National Comprehensive Cancer Network, 2021). In addition to chemotherapy, several targeted drugs have been approved, such as anti-VEGF antibody bevacizumab and multikinase inhibitor regorafenib. Overexpression of EGFR in CRC (Spano et al., 2005) provides a rationale for incorporation of targeted anti-EGFR drugs, such as cetuximab and panitumumab, into a chemotherapeutic regimen and significantly improved outcomes (Karapetis et al., 2008; Bokemeyer et al., 2009). However, only patients having a wild-type *KRAS*, *NRAS,* and *BRAF* genes can benefit from anti-EGFR therapy (Douillard et al., 2013). Among these *KRAS* is the most frequently mutated gene in CRC with a mutation frequency of 30%–40% (Siddiqi et al., 2009; Elsamany et al., 2014; Cárdenas-Ramos et al., 2017). Although *KRAS* mutation is a predictive biomarker for the anti-EGFR therapy of CRC patients, whether it is an independent prognostic factor in CRC is controversial (Deng et al., 2015; Zocche et al., 2015; Kim et al., 2016; Charlton et al., 2017). In a recent population-based (*n* = 8983) competing risk study, *KRAS* mutation indicated a poor prognosis of CRC patients (Dai et al., 2020). Considering its high occurrences, this makes *KRAS* one of the most important drug targets for CRC (Porru et al., 2018). Currently, several specific KRAS G12C inhibitors are being tested in clinical trials, including CRC patients (Nagasaka et al., 2020). However, despite multiple attempts in targeting mutant *KRAS*, it has proven to be difficult, and there is no approved drug for patients carrying a mutant-type of *KRAS* at present.

Notably, the KRAS gene frequently mutates in other cancers, such as pancreatic and lung, and is associated with poor prognosis, increased tumor aggressiveness and metastasis, and resistance to chemotherapy and targeted therapies (Slebos et al., 1990; Hanahan and Weinberg, 2011; Pylayeva-Gupta et al., 2011). Pancreatic ductal adenocarcinoma (PDAC) is the most common type of pancreatic cancer and is the third leading cause of adult cancer death with an extremely low survival rate (Ding and Billadeau, 2020). In PDAC, mutationally activated *KRAS* is found in more than 95% of cases. Exploration of PDAC vulnerabilities led to identification of glycogen synthase kinase-3β (GSK-3β) as a possible target. For the first time, the link between oncogenic KRAS mutation and GSK-3β overexpression was revealed in mouse PDAC cell lines expressing KRas^G12D^. Overexpression of constitutively active KRas enhanced GSK-3β promoter activity and increased mRNA expression (Zhang et al., 2011). Additional RNA interference GSK-3β specific inhibition studies revealed a critical role of GSK-3 in stimulating PDAC cell growth and antiapoptotic response. Moreover, a combination of GSK-3β inhibitors, such as AR-A014418 and 9-ING-41, with chemotherapeutic drugs had a synergistic effect in killing pancreatic cancer cells *in vitro* and *in vivo* in a xenograft model (Ding and Billadeau, 2020). GSK3 blockade is particularly efficient in growth inhibition of human tumors dependent on mutant KRas. In contrast mutant KRas-independent tumors do not require GSK-3β for viability, survival, and tumor growth (Kazi et al., 2018).

Regarding CRC, although KRAS mutations occur in about half of cases, they are probably not the primary initiating events (Porru et al., 2018). The degree to which these tumors depend on KRAS is still under investigation (McCormick, 2015), but GSK-3β is found to be overexpressed in many CRC cell lines (Shakoori et al., 2005). In addition, inhibition of GSK-3β decreased CRC tumor cell growth *in vitro* and *in vivo* (Shakoori et al., 2005, 2007). However, the effect of GSK-3β inhibition in combination with SoC chemotherapy on CRC growth has not been thoroughly investigated previously.

GSK-3 is a family of serine/threonine kinases represented by two isoforms, GSK-3α and GSK-3β, which share approximately 85% overall sequence homology. They have approximately 100 known targets and are involved in regulating normal cellular homeostasis and also tumor development, progression, and metastasis (Cormier and Woodgett, 2017). It was GSK-3β in the focus of the majority of the studies in the oncology field due to its known effects on many pathological processes (Pecoraro et al., 2021). GSK-3β regulates many biological pathways, including cyclic adenosine monophosphate (cAMP) signaling, Wnt, Hedgehog, Notch, transforming growth factor-beta (TGF-β), nuclear factor of activated T cells (NF-AT), and the agonists that act via stimulation of phosphatidylinositol 3-kinase (PI3K) (Cormier and Woodgett, 2017). GSK-3β is particularly important in tumor progression and modulation of oncogenes (including beta-catenin, cyclin D1, and c-Myc) because it acts as a part of the destruction complex of β-catenin in the Wnt/β-catenin pathway. Within this complex, GSK-3β phosphorylation of β-catenin results in β-catenin ubiquitin-mediated proteasomal degradation, thus preventing the transcriptional activation of the genes that are involved in cell proliferation and EMT (Wu and Pan, 2010). Surprisingly, GSK-3β activity is required for the growth of certain tumors (Shakoori et al., 2005; Ougolkov et al., 2006; Kazi et al., 2018). The underlying mechanisms of such multifaceted functions of GSK-3 are not fully understood. One of the possible explanations is the distinctive nuclear functions of this protein for tumor cells. GSK-3β is mainly considered to be a cytoplasmic protein but is found to be aberrantly accumulated in the nucleus in pancreatic cancer cell lines and human pancreatic adenocarcinomas. Moreover, GSK-3β positively regulates NF-κB activation and affects NF-κB–mediated survival and proliferation of cancer cells (Ougolkov et al., 2006). Another study reveals that inactivating GSK3α/β induced apoptosis in a β-catenin– and c-Myc–dependent manner (Kazi et al., 2018). Taken together, these findings suggest further investigation of the role of GSK-3β in cancer cells.

In the current study, we tested the combination of GSK-3 inhibitor 9-ING-41 and SoC drugs on CRC cell lines and primary CRC organoids with different *KRAS* mutation status. Among GSK-3β inhibitors that are currently tested in clinical trials, 9-ING-41 was selected as it was recently granted fast-track designation for treatment of patients with pancreatic cancer by the U.S. Food and Drug Administration (FDA). This small molecule was previously tested on several CRC cell lines harboring mutant and wild-type *KRAS* genes as a single drug and demonstrates superior growth inhibition activity toward oxaliplatin-resistant cells (Poloznikov et al., 2019; Huntington et al., 2021). Primary CRC organoids were selected due to their ability to preserve genetic and histopathological features of the original tumor and to predict clinical response (van de Wetering et al., 2015; Fujii et al., 2016; Tiriac et al., 2018; Nikulin et al., 2020). In addition, by means of transcriptome analysis, we sought to identify pathways involved in 9-ING-4–induced CRC cell growth inhibition.

## Materials and Methods

### Cell Culture

Human HT-29, RKO, and SW480 CRC cells were cultured in a complete cell culture medium consisting of DMEM high glucose (Gibco, United States) supplemented with 10% vol. fetal bovine serum (Gibco, United States), 1% vol. GlutaMax (Gibco, United States), and 1% vol. antibiotic-antimycotic solution (Gibco, United States). The cells were incubated in a cell culture incubator (37°C, 5% CO_2_). Subcultivation was performed every 2–3 days using trypsin-EDTA solution (PanEco, Russia). Cells were counted after trypan blue (Gibco, United States) staining using a Countess automated cell counter (Invitrogen, United States) according to the manufacturer’s protocol.

### Primary Patient Material

The organoid culture of CRC cells was established from resected metastatic tissue. Main clinical parameters of the three patients included in the study are summarized in Table 1. The study was approved by the local ethics committee.

**TABLE 1:** Clinical parameters of the patients included in the study.

Patient	Age	Sex	Localization of the metastatic lesion	Previous treatment
Patient 1	66	Female	Liver	XELOX, FOLFIRI + bevacizumab
Patient 2	54	Female	Lung	XELOX, FOLFOX + bevacizumab
Patient 3	45	Female	Lung	XELOX, FOLFIRI, Capecitabine

### Organoid Culture

To obtain primary organoid culture of CRC cells, resected metastatic tissue was used (Nikulin et al., 2020). The tissue sample was obtained during the examination of the surgically resected tissue block by a qualified pathologist who identified the resected tissue as a metastasis. Tissue was cut into small fragments and placed immediately into MACS tissue storage solution (Miltenyi Biotec, Germany). The sample was stored for several hours at 4°C. Then, the tissue fragments were transferred into a tube for tissue homogenization (gentleMACS C Tube, Miltenyi Biotec, Germany), and the enzyme cocktail from the Tumor Dissociation Kit Human (Miltenyi Biotec, Germany), which consisted of 2.2 ml of DMEM/F-12 culture medium (Thermo Fisher Scientific, United States), 100 μL of Enzyme H solution (Miltenyi Biotec, Germany), 50 μL of Enzyme R solution (Miltenyi Biotec, Germany), and 12.5 μL of Enzyme A solution (Miltenyi Biotec, Germany), was added to the same tube. Then, the tube was tightly closed with a lid and placed into a gentleMACS Octo Dissociator (Miltenyi Biotec, Germany). For tissue dissociation, the “37C_h_TDK_3” program was used. After the end of the program, the tube was removed from the dissociator. The resulting suspension was centrifuged at 300 g for 10 min. The supernatant was removed, and the pellet was resuspended in 10 ml of DPBS (Thermo Fisher Scientific, United States). Then, the suspension was recentrifuged with the same parameters, the supernatant was also removed, and the pellet was resuspended in DMEM/F-12 culture medium (Thermo Fisher Scientific, United States). Then, the tube with the suspension was placed on ice, and the suspension was mixed with Matrigel Growth Factor Reduced (GFR) Basement Membrane Matrix (Corning, United States) in the ratio 1:2. Then, 50 μL drops of the resulting suspension in the extracellular matrix were transferred into the wells of a 24-well culture plate (TPP, Switzerland) and placed into a cell culture incubator (37°C, 5% CO_2_) for 20 min for solidification of the gel. Cell density in the resulting suspension in the extracellular matrix was 400,000 cells/ml. Then, 750 μL of complete cell culture medium was added to each well, and the plate was incubated in a cell culture incubator. The recipe of the complete cell culture medium for CRC organoids is based on previously published data with minor modifications (van de Wetering et al., 2015; Driehuis et al., 2020; Dekkers et al., 2021). The complete cell culture medium consisted of Advanced DMEM/F12 (Gibco, United States) supplemented with 1% vol. GlutaMax (Gibco, United States), 1% vol. antibiotic-antimycotic solution (Gibco, United States), 1% vol. HEPES (Gibco, United States), 2% vol. B27 (Gibco, United States), 1.25 mM n-Acetyl Cysteine (Sigma, United States), 10 mM Nicotinamide (Sigma, United States), 250 ng/ ml R-spondin 1 (PeproTech, United States), 100 ng/ ml Noggin (PeproTech, United States), 50 ng/ ml human EGF (Gibco, United States), 10 nM Gastrin I (Sigma, United States), 500 nM A83-01 (STEMCELL Technologies, Canada), 1 uM SB202190 (Tocris Bioscience, United Kingdom), 10 nM Prostaglandine E2 (Sigma, United States), and 5 uM Y-27632 (STEMCELL Technologies, Canada). Cell culture medium was replaced every 48 h. Cells were inspected visually by an inverted Primo Vert microscope (Carl Zeiss, Germany). Organoids were subcultured (1:5 dilution) every 2 weeks with the help of TrypLE Express (Thermo Fisher Scientific, United States).

### Histology

Fragments of the original tumor tissue were fixed in 10% neutral buffered formalin (overnight at room temperature) and embedded in paraffin. Formalin-fixed samples of tumor organoids were covered with Histogel (Thermo Fisher Scientific, United States) and then embedded into paraffin. Serial sections with a thickness of 4 μm were cut and then were routinely stained with hematoxylin-eosin and then examined by light microscopy.

### DNA Sequencing

Next-generation sequencing of the DNA isolated from the tumor tissue and organoids was carried out with the Oncomine Comprehensive Assay V3 (Thermo Fisher Scientific, United States) based on the Ion Torrent S5 System (Thermo Fisher Scientific, United States). Snap-frozen tumor tissue and organoids were used for isolation of DNA. The alignment was carried out on the basis of the GRCh37 genomic assembly. Single nucleotide variants (SNV) and small insertions and deletions (indel) as well as larger genome rearrangements were identified by Atlas Oncology Diagnostics (Ivanov et al., 2019).

### RNA Sequencing and RT-PCR

To detect more pronounced changes in mRNA levels, organoids and cell lines were treated with 9-ING-41 for 24 h (Duffy et al., 2014). Then, cells were lysed with the QIAzol Lysis Reagent (Qiagen, Germany). The lysates were stored at −80°C before RNA isolation. RNA isolation was performed using miRNeasy Micro Kit (Qiagen, Germany) according to the manufacturer’s protocol. Nanodrop ND-1000 (Thermo Fisher Scientific, United States) was used to assess quantity and purity of the extracted RNA. Total RNA samples were also QC-checked using an Agilent 2100 Bioanalyzer (Agilent Technologies, United States). For each group, three independently obtained samples of RNA were used.

Libraries for mRNA sequencing were prepared from total RNA samples using an Illumina Stranded mRNA Library Prep Kit (Illumina, United States). Each sample was sequenced on the NextSeq 550 (Illumina, United States) to generate paired-end, 75-nucleotide reads.

Real-time PCR was used to assess changes in the expression of selected individual genes. Reverse transcription of RNA was performed using the MMLV RT kit (Evrogen, Russia) according to the manufacturer’s protocol. The obtained cDNA samples were stored at −20°C. qPCRmix-HS SYBR (Evrogen, Russia) was used for RT-PCR performed with DTprime detecting amplifier (DNA Technology).

The oligonucleotide primers used for RT-PCR were designed based on the mRNA sequences of the studied genes from the UCSC Genome Browser database (Kent et al., 2002). Primer selection was performed using Primer-BLAST software (Ye et al., 2012). *ACTB* and *GAPDH* were selected as reference genes. The sequences of the primers used for RT-PCR are presented in Table 2. The evaluation of the differences in the expression of the selected genes resulting from the addition of 9-ING-41 in comparison with the control cells was carried out using the software REST 2009 v.2.0.13 (Pfaffl et al., 2002; Vandesompele et al., 2002).

**TABLE 2:** Oligonucleotide primers for RT-PCR.

Gene	Sequences
UBE2C	Forward:
	5′- AAA​GTG​GTC​TGC​CCT​GTA​TGA-3′
	Reverse:
	5′- GCA​TGT​GTG​TTC​AAG​GGA​CT-3′
MMP1	Forward:
	5′-TTT​GCC​GAC​AGA​GAT​GAA​GTC​CG-3′
	Reverse:
	5′-AGG​GAA​GCC​AAA​GGA​GCT​GTA​GA-3′
TUBB	Forward:
	5′-CTG​GAC​CGC​ATC​TCT​GTG​TAC​TAC-3′
	Reverse:
	5′-GAC​CTG​AGC​GAA​CAG​AGT​CCA​T-3′
CDK1	Forward:
	5′-AGG​GTA​GTC​TGG​TCT​TTC​TTT​GGC​T-3′
	Reverse:
	5′-CAC​CTA​CAA​CCA​CCA​CTC​TGC​C-3′
ACTB	Forward:
	5′-CTG​GAA​CGG​TGA​AGG​TGA​CA-3′
	Reverse:
	5′-AAG​GGA​CTT​CCT​GTA​ACA​ACG​CA-3′
GAPDH	Forward:
	5′-GAA​GGT​GAA​GGT​CGG​AGT​C-3′
	Reverse:
	5′-GAA​GAT​GGT​GAT​GGG​ATT​TC-3′

### Bioinformatic Analysis

The quality of FASTQ files was assessed with FastQC v0.11.9 (Babraham Bioinformatics, United Kingdom) and multiQC v1.9 (Ewels et al., 2016). The adapters were trimmed with fastp 0.21.1 (Chen et al., 2018). The trimmed mRNA-seq reads were mapped on the reference human genome GENCODE release 37 (GENCODE GRCh38. primary assembly) with STAR 2.7.7a (Dobin et al., 2013). GENCODE release 37 genome annotation (gencode.v37. primary assembly. annotation) (Frankish et al., 2019) was used to generate the count matrix with the featureCount tool from ssubread-2.0.1 aligner (Liao et al., 2013, 2014).

Differential expression analysis was conducted using DESeq2 v1.28.1 (Love et al., 2014). False discovery rates (FDRs) were calculated by the Benjamini–Hochberg procedure. To assess the statistical significance of differences in gene expression, FDR *p*-values with threshold level of .05 were used. To construct heat maps of gene expression, regularized-logarithm transformation of counts was used (Love et al., 2014). Only differentially expressed genes (FDR *p*-values < .05) were included in the heat maps.

Gene set enrichment analysis was performed with GSEA 4.1.0 (Mootha et al., 2003; Subramanian et al., 2005). The Hallmark (Liberzon et al., 2015), Canonical pathways (includes gene sets from BIOCARTA (Nishimura, 2001), KEGG (Kanehisa, 2000), PID (Schaefer et al., 2009), REACTOME (Jassal et al., 2019) and WikiPathways (Martens et al., 2021) pathway databases), GTRD subset of TFT (Kolmykov et al., 2021), miRDB subset of MIR (Chen and Wang, 2020), and Oncogenic signature gene sets were included in the analysis (Liberzon et al., 2011). To assess the statistical significance of gene set enrichment, FDR *p*-values with a threshold level of .05 were used.

### Drug Test

HT-29, RKO, and SW480 cells were seeded into 96-well plates (TPP, Switzerland) in 100 ul of complete cell culture medium (5000 per well). Organoids were diluted in Matrigel GFR Basement Membrane Matrix (Corning, United States) and seeded into 96-well plates (TPP, Switzerland) (50 organoids per well). After solidification of the gel, 100 ul of complete cell culture medium was added into each well.

After 24 h, the cell culture medium was replaced with medium containing single SoC drugs and SoC drug combinations with or without 9-ING-41. Clinically relevant concentrations were used in the assay: 25 uM for 5-FU (Sigma, United States) (Larsson et al., 1996), 10 uM for Oxaliplatin (Medac, Germany) (Graham et al., 2000), and 2 uM for 9-ING-41 (Ugolkov et al., 2016; Kuroki et al., 2019). 9-ING-41 was synthesized and tested according to a previously published procedure (Gaisina et al., 2009). Stock solutions of 5-FU and 9-ING-41 were prepared in DMSO; stock solution of Oxaliplatin was prepared in water (Hall et al., 2014). The treatments were compared with vehicle controls containing the same quantity of DMSO and water.

Then cells were incubated for 3 h in a cell culture incubator (37°C, 5% CO_2_), and the medium was replaced with fresh complete cell culture medium. Then, plates were incubated in a cell culture incubator (37°C, 5% CO_2_) for 72 h. The relative number of viable cells was measured with the MTS assay CellTiter 96 Aqueous One Solution Cell Proliferation Assay kit (Promega, United States) according to the manufacturer’s instructions (Steele et al., 2019). Growth rate of cancer cells was calculated as described previously (Hafner et al., 2016).

Each experiment was performed three times for cell lines and five times for organoids; three technical replicates were used in each experiment. Two-way ANOVA with Tukey *post hoc* test was used to assess changes in the growth rate of cancer cells in the drug tests. The first factor was SoC treatment with the levels corresponding to different SoC drugs and combinations. The second factor was 9-ING-41 (presence or absence of 9-ING-41). The differences were considered statistically significant if adjusted *p*-values were less than .05.

## Results

### Patient-Derived Tumor Organoids Share Morphologic Features with Clinical Tumors

**FIGURE 1:**
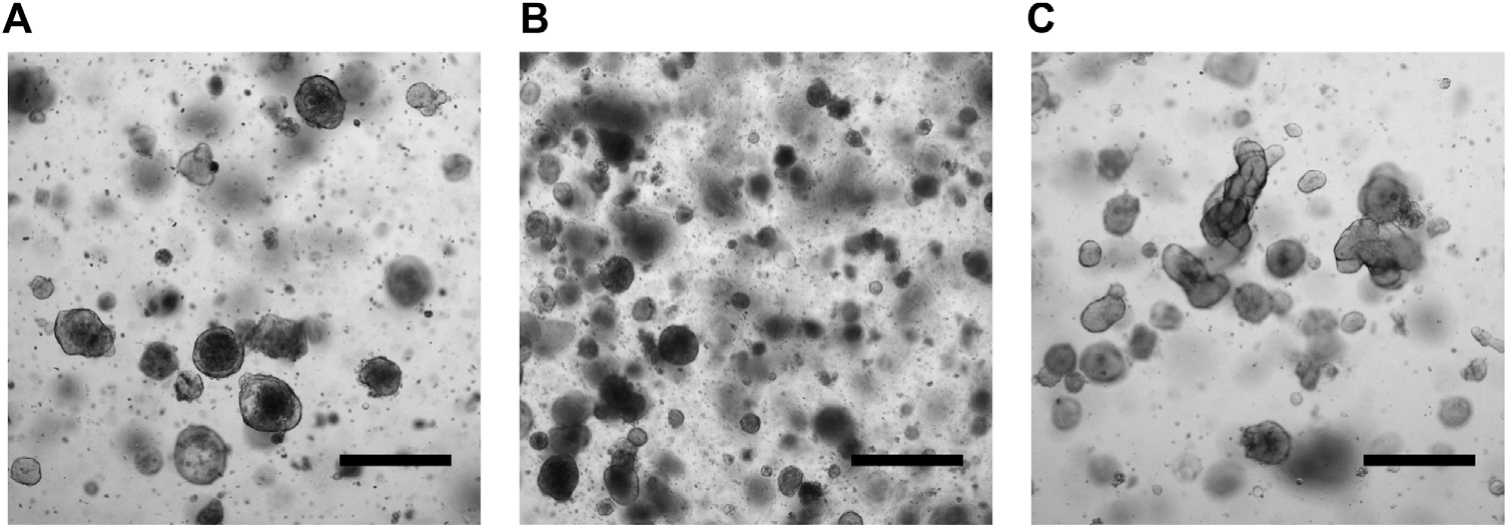
Micrographs of CRC PDOs. Patient 1 **(A)**. Patient 2 **(B)**. Patient 3 **(C)**. Scale bars indicate 500 μm.

Patient-derived tumor organoids (PD-TOs) were generated starting from digested tissue, embedded in 50 μL of Matrigel, and cultured for a maximum of 3 weeks without passaging. After subculturing, we observed extensive cell growth starting from the second day of the seeding (Figure 1).

**FIGURE 2:**
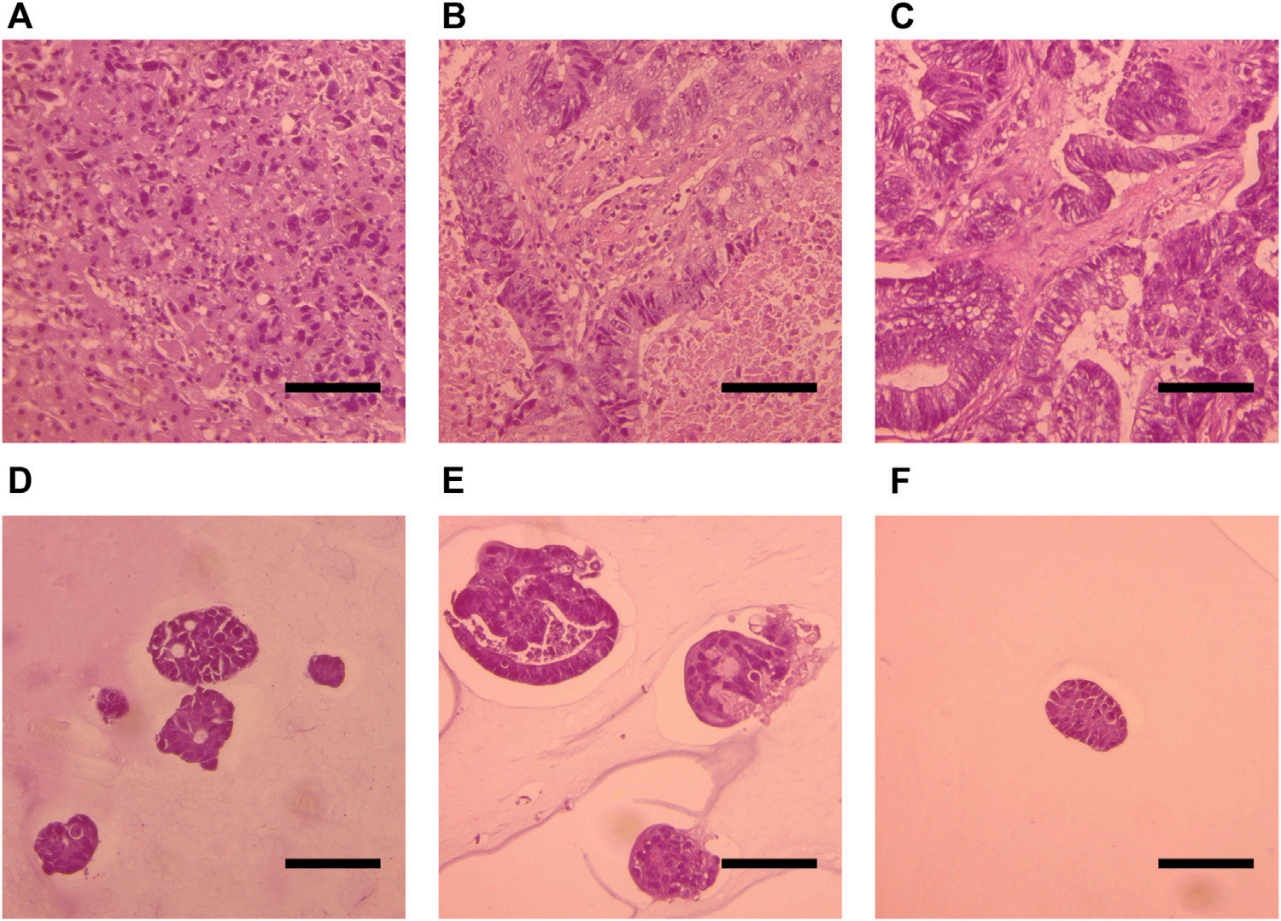
Histological analysis of H&E-stained slides of the primary tumor **(A,B,C)** and organoids **(D,E,F)**. Patient 1 **(A, D)**. Patient 2 **(B,E)**. Patient 3 **(C, F)**. Scale bars indicate 100 μm.

To determine whether the generated organoids consist of CRC cells rather than normal epithelial cells, we performed H&E histologic analysis of primary tumor organoids. An experienced pathologist compared cell morphology in organoids to the primary tumors and confirmed that all generated organoids cultures consist of malignant cells (Figure 2).

### Tumor Organoids Capture Tumor Heterogeneity

**FIGURE 3:**
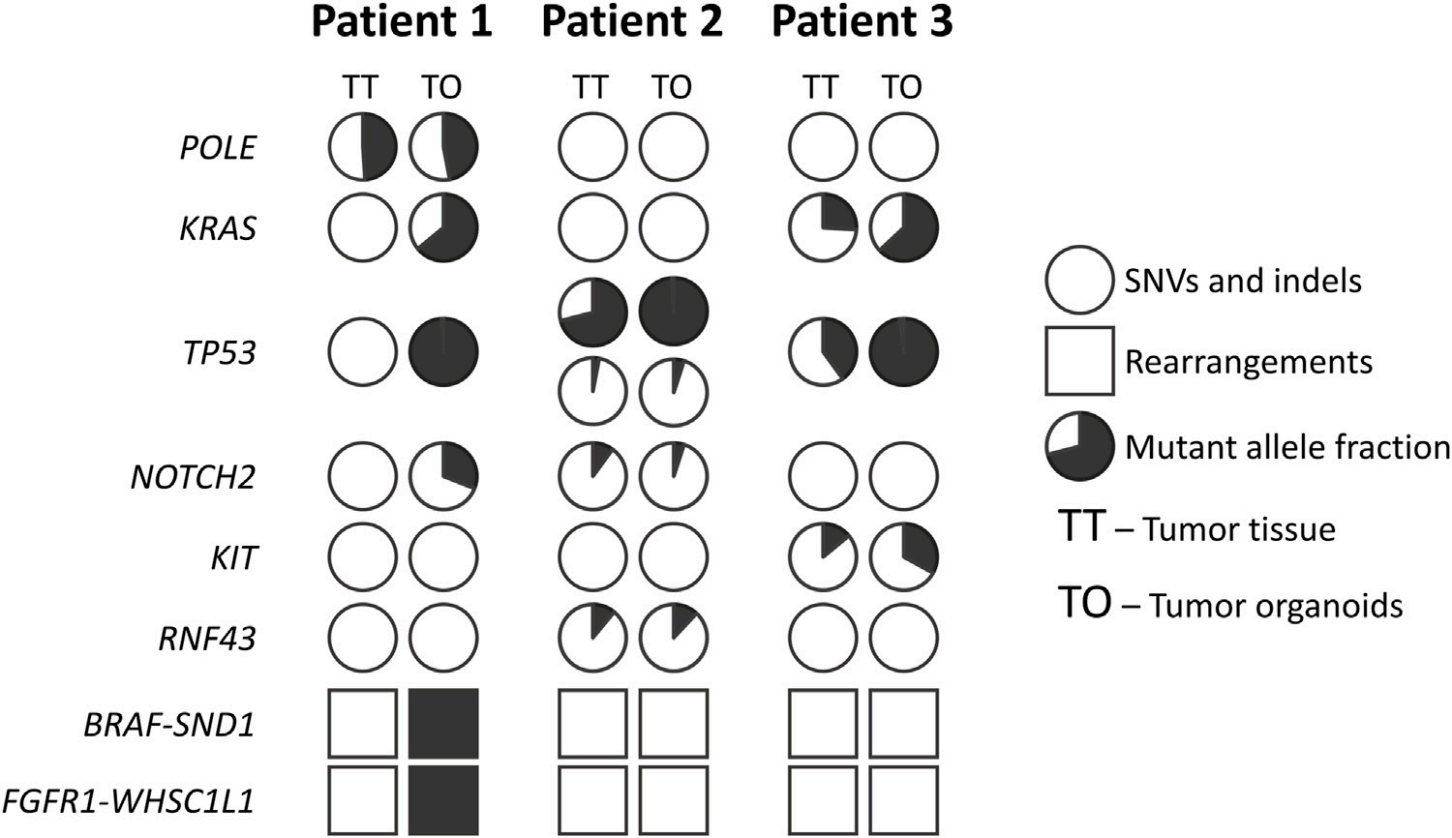
Overview of the mutations found in the tumor organoids and tumor tissue samples. Pie charts indicate percentage of mutant allele in the sample.

The analysis of mutations in tumor tissue and tumor organoids (Figure 3, Supplementary Table S1) revealed that the initial tissue from liver metastasis of Patient 1 contained only one SNV in the *POLE* gene (p.F990S). This variant was not found in the Catalogue of Somatic Mutations in Cancer and most probably represents a germline mutation (Tate et al., 2019). In the corresponding tumor organoids, in addition to the same SNV in the *POLE* gene, several other point mutations were identified: *KRAS* (p.G12D), *TP53* (p.V173A), and *NOTCH2* (p.R2051Q). Moreover, two large genome rearrangements were also detected; the first one involved *BRAF* and *SND1* genes, and the second one involved *FGFR1* and *WHSC1L1* genes.

Lung metastasis tissue from Patient 2 contained two different mutations in the *TP53* gene (p.C135Y and p. C135S) and single mutations in *NOTCH2* (p.A3F) and *RNF43* (p.R117S) genes. These mutations were conserved in tumor organoids. No large genome rearrangements were found in tumor tissue as well as in tumor organoids from Patient 2.

Initial metastatic tumor tissue from Patient 3 contained the following mutations: *KRAS* (p.G12C), *TP53* (p.P177R), and KIT (p.A621T). The same mutations were found in the corresponding tumor organoids. Neither initial tumor tissue nor tumor organoids from Patient 3 contained large genome rearrangements.

### 9-ING-41 Inhibits Growth of Colorectal Tumor Cells

**FIGURE 4:**
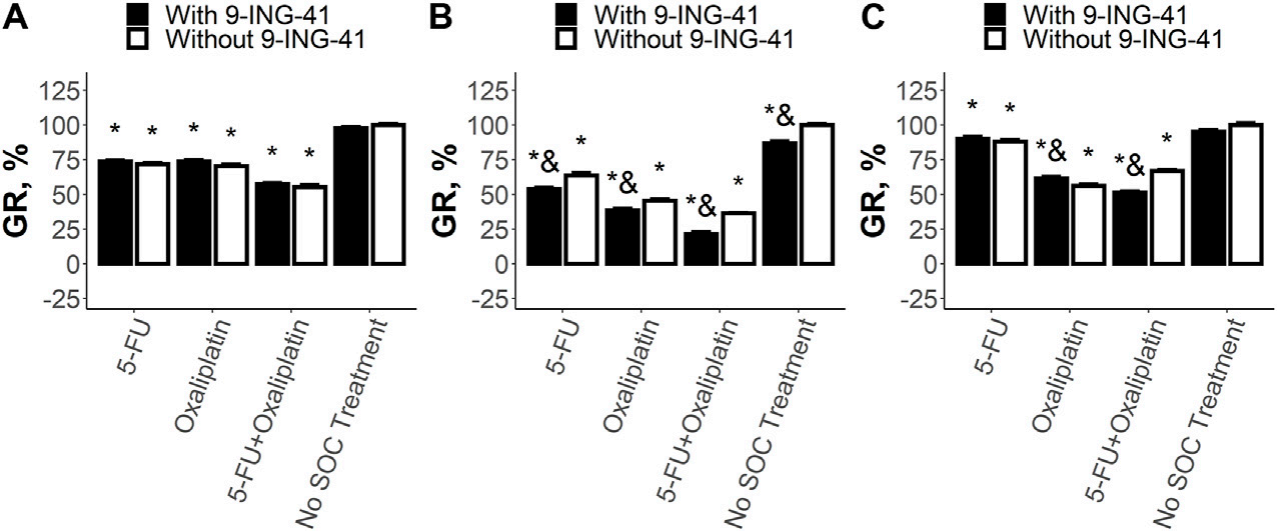
Results of the drug test for the tested SoC drugs (5-FU and Oxaliplatin) and their combination on HT-29 **(A)**, RKO **(B)**, and SW480 **(C)** cells. Error bars represent standard error of mean (SEM). Each experiment has been performed three times. *–*p* < .05 versus control (no SoC treatment without 9-ING-41), and–*p* < .05 versus corresponding treatment without 9-ING-41.

Analysis of the sensitivity of CRC cell lines to the SoC drugs and 9-ING-41 demonstrated various types of response (Figure 4). The growth inhibitory effect of the single SoC drugs and their combination was statistically significant (*p* < .05) for all tested cell lines and ranged from 12% to 64%. On the other hand, response to 9-ING-41 was more heterogenous. For example, HT-29 cells were resistant to the transient inhibition of GSK-3β by 9-ING-41 (Figure 4A). Moreover, 9-ING-41 did not improve the results of the SoC drugs (*p* > .05) in this case. In contrast, RKO cells (Figure 4B) were sensitive to 9-ING-41 (*p* < .05), and it significantly enhanced the growth inhibitory effect of 5-FU (*p* < .05), oxaliplatin (*p* < .05), and their combination (*p* < .05). SW480 cells (Figure 4C) were resistant to pure 9-ING-41; however, the growth inhibitory effect of the combination of 5-FU and oxaliplatin was more pronounced in the presence of 9-ING-41 (*p* < .05).

**FIGURE 5:**
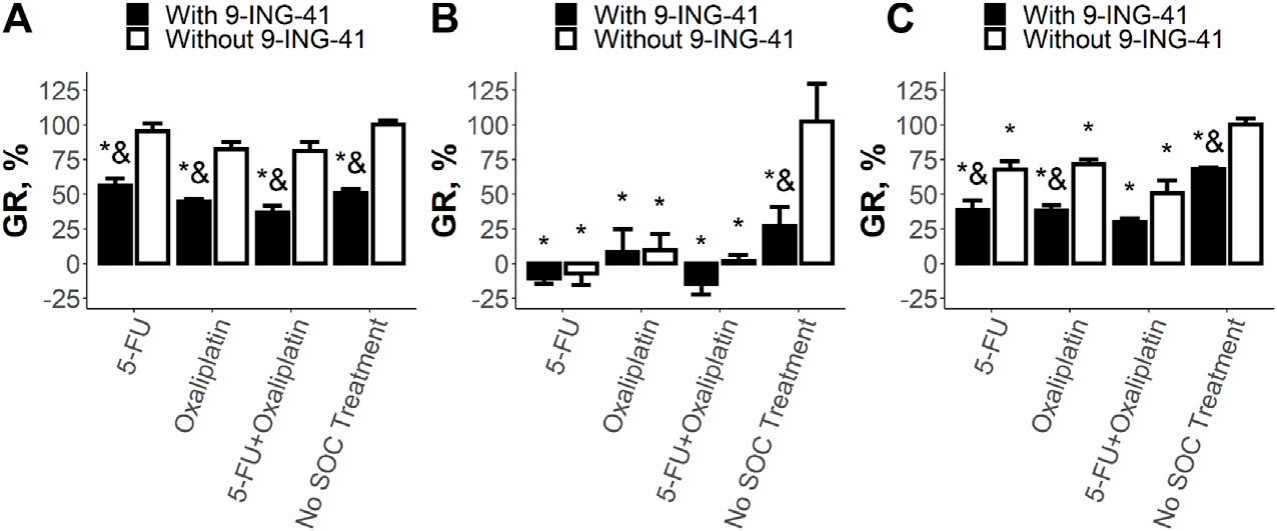
Results of the drug test for the tested SoC drugs (5-FU and Oxaliplatin) and their combination on the organoids from Patient 1 **(A)**, Patient 2 **(B)**, and Patient 3 **(C)**. Error bars represent standard error of mean (SEM). Each experiment has been performed five times. *–*p* < .05 versus control (no SoC treatment without 9-ING-41), and–*p* < .05 versus corresponding treatment without 9-ING-41.

Overall, all tested patient-derived tumor organoids were more sensitive to 9-ING-41. For Patient 1, analysis of the results of the drug tests (Figure 5A) revealed that clinically relevant concentrations (Larsson et al., 1996; Graham et al., 2000) of neither 5-FU (*p* = .99) nor Oxaliplatin (*p* = .17) significantly inhibited growth of PD-TOs. The combination of these two drugs was also ineffective (*p* = .1). In contrast, 9-ING-41 markedly reduced the growth rate of the CRC organoids from Patient 1 by 44%–62% both alone (*p* < .05) and in combination with the tested SoC drugs (*p* < .05). Moreover, addition of 9-ING-41 to the tested single SoC drugs as well as to their combination always led to better results in comparison with the corresponding treatment without 9-ING-41 (reduction of the growth rate by 38%–43%, *p* < .05). However, the magnitude of the effect of the combinations of 9-ING-41 with the SoC drugs did not differ statistically significant from pure 9-ING-41, suggesting that the main effect in this case is produced by 9-ING-41 rather than SoC drugs.

For Patient 2, it was shown (Figure 5B) that both 5-FU and Oxaliplatin as well as their combination almost completely stopped the growth of cancer cells (*p* < .05). Addition of 9-ING-41 to these treatment variants did not change significantly the growth rate (*p* = 1 for 5-FU and Oxaliplatin, *p* = .99 for 5-FU + Oxaliplatin). Interestingly, in this particular case, single 9-ING-41 also significantly reduced the growth rate (*p* < .05), and its effect was indistinguishable from the SoC treatment (*p* = .66 for 5-FU, *p* = .98 for Oxaliplatin, and *p* = .89 for 5-FU + Oxaliplatin) as well as from the combination of SoC treatment with 9-ING-41 (*p* = .54 for 5-FU, *p* = .98 for Oxaliplatin, and *p* = .42 for 5-FU + Oxaliplatin).

For Patient 3, the drug test revealed (Figure 5C) that both 5-FU (*p* < .05) and Oxaliplatin (*p* < .05) as well as their combination (*p* < .05) significantly reduced the growth rate of colorectal tumor organoids by 28%–49%. 9-ING-41 alone, in this case, was also active and decreased the growth rate by 32% (*p* < .05). Moreover, the effect of pure 9-ING-41 was not significantly different from the SoC treatment variants (*p* = 1 for 5-FU and Oxaliplatin and *p* = .35 for 5-FU + Oxaliplatin). Interestingly, addition of 9-ING-41 to the single SoC drugs 5-FU and Oxaliplatin improved the effect of the treatment by around 30% (*p* < .05 and *p* = .05 respectively). In both cases, the growth inhibitory effect of the combinations containing 9-ING-41 was significantly better than the effect of pure 9-ING-41 (*p* < .05 for both 5-FU and Oxaliplatin). All these data suggest the presence of the additional therapeutic effect between the single SoC drugs and 9-ING-41. In the case of combination of 5-FU and Oxaliplatin, the benefit from 9-ING-41 (around 20% reduction in the growth rate) did not reach statistical significance (*p* = .14), but the effect of the combination was significantly better than the effect of 9-ING-41 alone (*p* < .05).

Overall, our data demonstrate that the response of colorectal cell lines to transient action of 9-ING-41 can vary. In some cases, it can be active alone, and sometimes it can significantly enhance the growth inhibitory effect of the SoC drugs. On the other hand, in all analyzed samples of tumor organoids, 9-ING-41 was active and decreased the growth rate of CRC cells. Its efficiency was either significantly higher than one of the SoC drugs (Patient 1) or comparable to it (Patients 2 and 3). Moreover, in case of intermediate (Patient 3) or low (Patient 1) sensitivity to the SoC drugs, addition of 9-ING-41 can markedly improve the results of the standard treatment.

To elucidate molecular mechanisms underlying the ability of 9-ING-41 to overcome chemotherapy resistance in CRC cells, we carried out a transcriptome analysis of the most resistant organoids derived from Patient 1.

### Transcriptomic Analysis

Overall, transcriptomic analysis revealed (Supplementary Table S2) that, after treatment with 9-ING-41 for 24 h, 325 genes significantly changed their expression (FC > 1.5, FDR *p*-value < .05): 155 were upregulated, and 170 were downregulated. Further GSEA showed that 280 gene sets were significantly upregulated, and 118 gene sets were significantly downregulated after treatment with 9-ING-41 (Supplementary Table S3).

**FIGURE 6:**
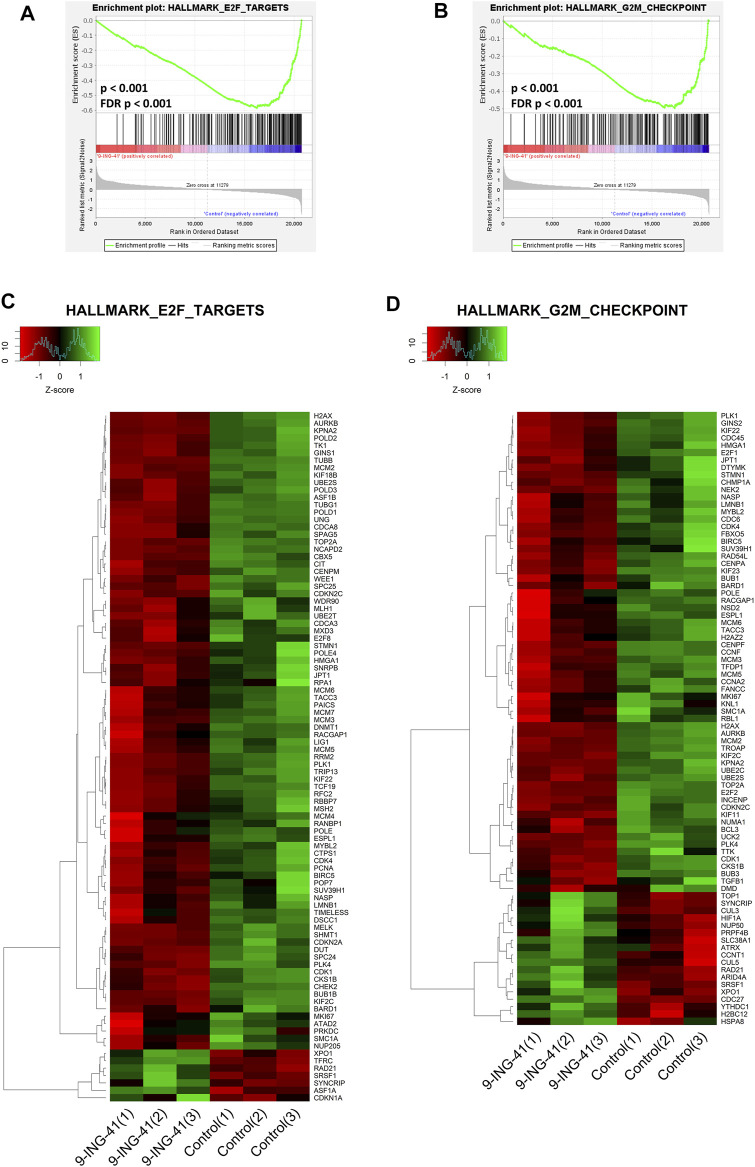
GSEA enrichment plots **(A,B)** and heat maps **(C,D)** for hallmark E2F targets **(A,C)** and hallmark G2M checkpoint **(B,D)** gene sets.

The top enriched downregulated gene set was HALLMARK E2F TARGETS (FDR *p* < .001), containing the genes encoding cell cycle–related targets of E2F transcription factors (Figures 6A,C). Top downregulated genes from this gene set were TUBB (FC = −2.4, FDR *p* < .001), SPC24 (FC = −2.0, FDR *p* < .001), and AURKB (FC = −1.9, FDR *p* < .001). E2F together with CDK and RB proteins form the key transcription machinery for cell cycle progression (Kent and Leone, 2019). Interestingly, a lot of other gene sets related to E2F were also significantly downregulated. This list includes E2F3 UP.V1 UP (FDR *p* = .008), PID E2F PATHWAY (FDR *p* = .01), reactome transcription of e2f targets under negative control by dream complex (FDR *p* = .02), reactome transcription of E2F targets under negative control BY P107 RBL1 AND P130 RBL2 in complex with HDAC1 (FDR *p* = .02), and E2F1 UP.V1 UP (FDR *p* = .03). Moreover, the gene set WP RETINOBLASTOMA GENE IN CANCER (FDR *p* < 0.001) was among the top five downregulated ones. Also worth noting is that the gene sets produced previously in the experiments on knockout of RB in mice keratinocytes (Lara et al., 2008) were also significantly downregulated: RB DN. V1 UP (FDR *p* = .001), RB P107 DN. V1 UP (FDR *p* < .001), RB P130 DN. V1 UP (FDR *p* = .02).

**FIGURE 7:**
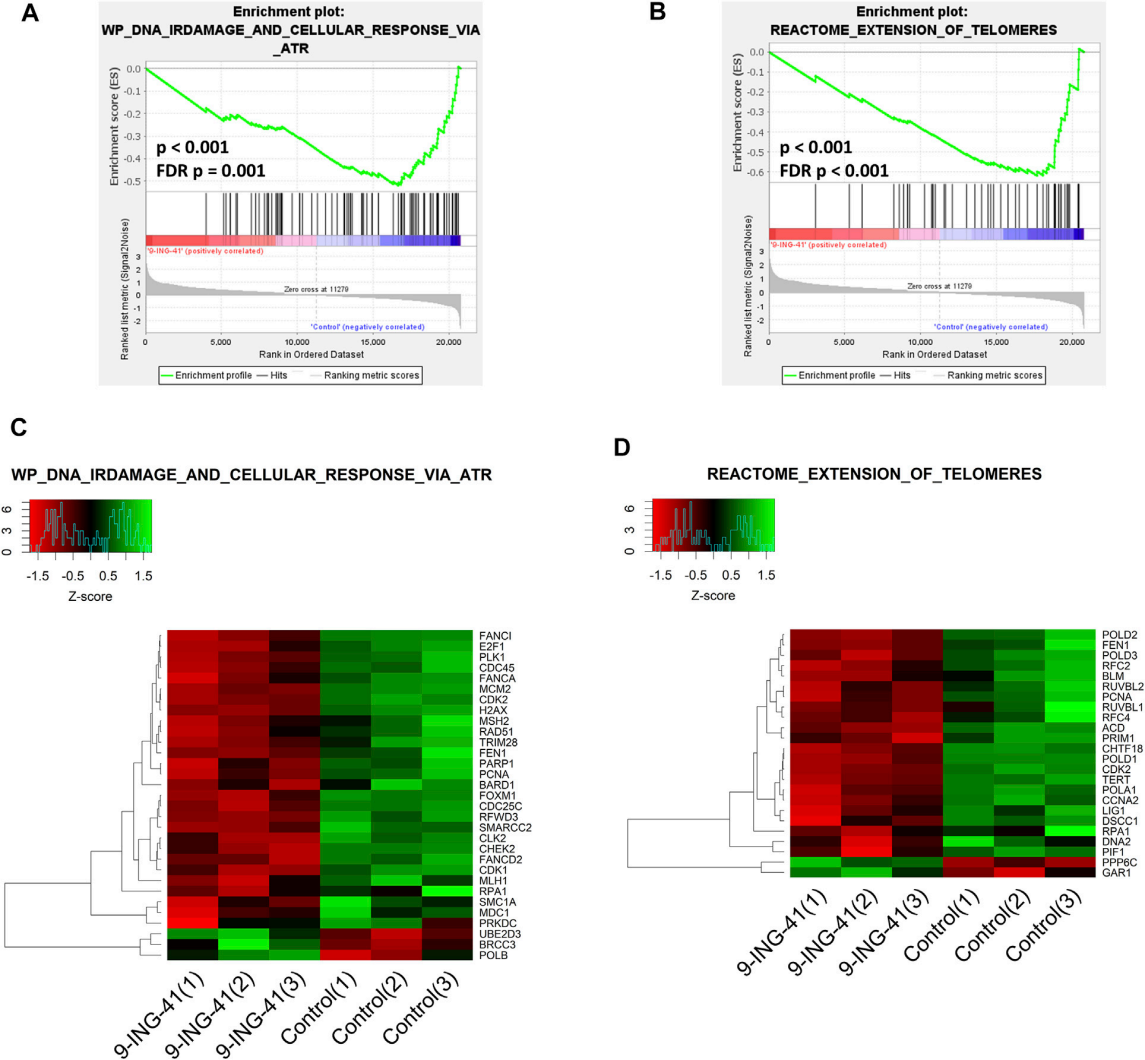
GSEA enrichment plots **(A,B)** and heat maps **(C,D)** for Wp DNA irdamage and cellular response *via* atr **(A,C)** and reactome extension of telomeres **(B,D)** gene sets.

In addition to the CDK-RB-E2F axis, a lot of other gene sets, related to the cell cycle were enriched in control cells in comparison to the treated cells, indicating global suppression of cell cycle progression by 9-ING-41 (Table 3), including hallmark G2M checkpoint (Figures 6B,D), reactome G1 s specific transcription, and reactome mitotic prometaphase. Interestingly, a number of other gene sets connected to DNA repair and telomere maintenance were also suppressed after treatment with 9-ING-41 (Figure 7; Table 4).

**TABLE 3:** Selected cell cycle–related gene sets downregulated after treatment with 9-ING-41. NES–Normalized Enrichment Score.

Gene set	NES	FDR p-value
Hallmark G2M checkpoint	−2.9	< .001
Reactome G1 s specific transcription	−2.4	< .001
Reactome mitotic prometaphase	−2.3	< .001
Kegg dna replication	−2.3	< .001
WP dna replication	−2.3	< .001
Reactome dna replication	−2.3	< .001
Reactome s phase	−2.1	.002
Reactome G0 and early G1	−2.1	.002
Reactome activation of the pre-replicative complex	−2.1	.002
Reactome separation of sister chromatids	−2.1	.003
Reactome mitotic G1 phase and G1 s transition	−2.1	.003
Reactome recruitment of numa to mitotic centrosomes	−2.1	.003
Wp G1 to s cell cycle control	−2.1	.004
Wp cell cycle	−2.0	.004
Reactome mitotic spindle checkpoint	−2.0	.004
Reactome mitotic metaphase and anaphase	−2.0	.008
Reactome cyclin a b1 b2 associated events during G2 M transition	−2.0	.009

**TABLE 4:** Selected DNA repair and telomere maintenance–related gene sets downregulated after treatment with 9-ING-41. NES–Normalized Enrichment Score.

Gene set	NES	FDR p-value
Reactome telomere c strand lagging strand synthesis	−2.4	< .001
Reactome extension of telomeres	−2.4	< .001
Wp dna repair pathways full network	−2.3	< .001
Reactome polymerase switching on the c strand of the telomere	−2.3	< .001
Wp base excision repair	−2.3	< .001
Reactome resolution of abasic sites ap sites	−2.3	< .001
Wp dna mismatch repair	−2.4	< .001
Reactome resolution of ap sites via the multiple nucleotide patch replacement pathway	−2.2	< .001
Reactome pcna dependent long patch base excision repair	−2.2	< .001
Reactome gap filling dna repair synthesis and ligation in gg ner	−2.2	< .001
Wp dna irdamage and cellular response *via* atr	−2.2	< .001
Reactome processive synthesis on the c strand of the telomere	−2.2	< .001
Kegg base excision repair	−2.1	.002
Reactome mismatch repair	−2.1	.003
Reactome activation of atr in response to replication stress	−2.1	.004
Pid atr pathway	−1.8	.02
Biocarta atrbrca pathway	−1.8	.02

**FIGURE 8:**
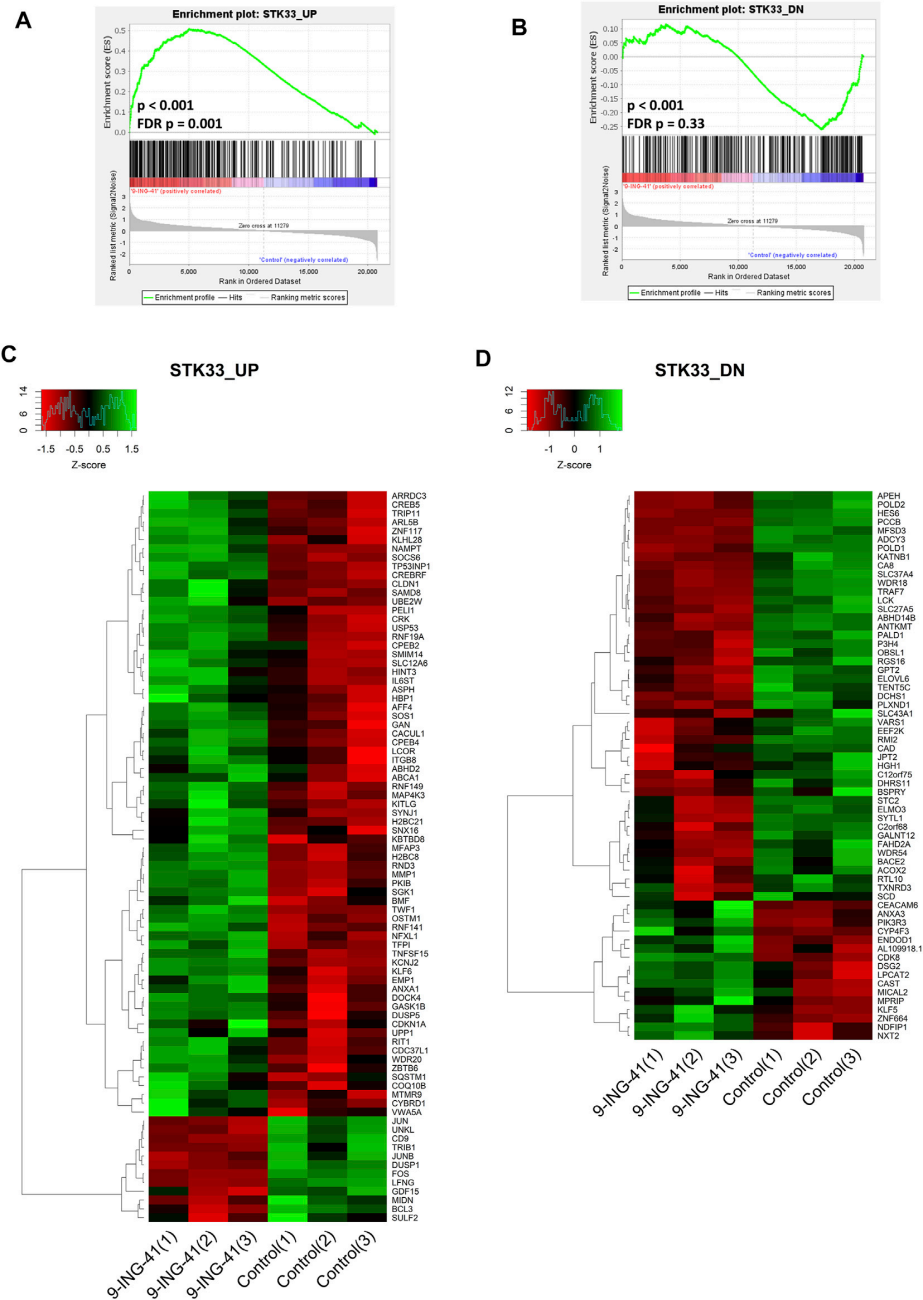
GSEA enrichment plots **(A,B)** and heat maps **(C,D)** for STK33 UP **(A,C)** and STK33 DN **(B,D)** gene sets.

The top enriched upregulated gene set was STK33 SKM UP (FDR *p* = 0.002), containing the genes upregulated in SKM-1 acute myeloid leukemia (AML) cells after knockdown of STK33. Another two gene sets related to STK33, named STK33 NOMO UP (FDR *p* = .001, contains the genes upregulated in NOMO-1 AML cells after knockdown of STK33) and STK33 UP (FDR *p* = .001, combines two previously mentioned gene sets) were also in the top five list of the enriched upregulated gene sets (Figures 8A,C). The most significantly regulated genes from the gene set STK33 UP were *MMP1* (FC = 2.4, FDR *p* < .001), *H2BC8* (FC = 2.3, FDR *p* < .001) and *CYBRD1* (FC = 7.2, FDR *p* = .048). Interestingly, the STK33 DN gene set, which consisted of the genes that were downregulated in NOMO-1 and SKM-1 cells after knockdown of STK33, was also downregulated after treatment with 9-ING-41 (Figures 8B,D), but the changes were insignificant after correction for multiple comparisons (*p* < .001, FDR *p* = .33). *STK33* is a gene located in human chromosomal region 11p15 and codes a serine/threonine kinase (Mujica et al., 2001). It is shown that knockdown of *STK33* leads to a decrease in viability of KRAS mutant cancer cells (Scholl et al., 2009). Thus, our results suggest that action of 9-ING-41 can have similarities with suppression of *STK33.*

**FIGURE 9:**
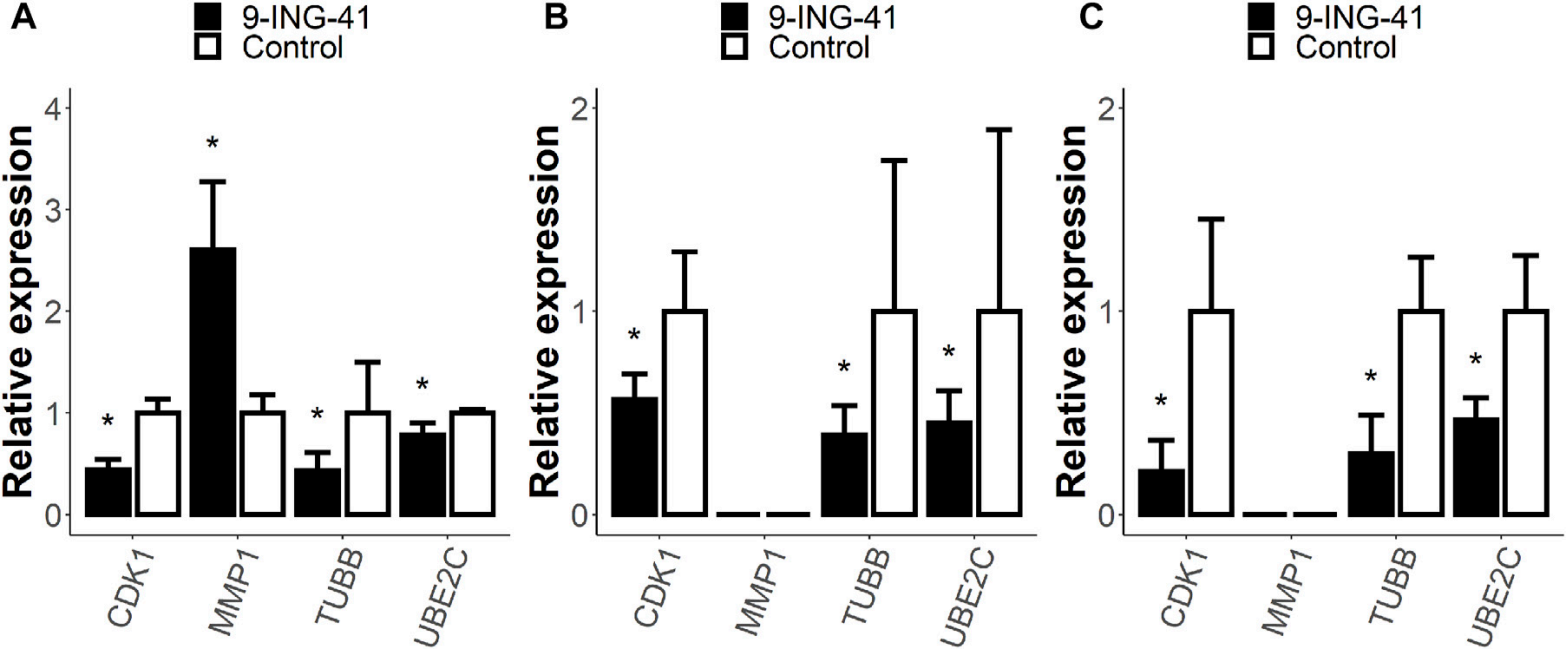
Relative expression of selected genes from enriched gene sets measured with RT-PCR. Patient 1 organoids **(A)**, HT-29 cells **(B),** and RKO cells **(C)**. Error bars represent 95% confidence intervals. The expression of the MMP1 gene was too low and was not measured in HT-29 and RKO cells. *–*p* < .05 versus control.

To verify the results of the RNA-seq, we performed RT-PCR for selected genes from the enriched gene sets (Figure 9). We chose *TUBB* gene (RNA-seq FC = −2.4, FDR *p* < .05) from the HALLMARK E2F TARGETS gene set, *UBE2C* gene (RNA-seq FC = −1.7, FDR *p* < .05) from the HALLMARK G2M CHECKPOINT gene set, *CDK1* gene (RNA-seq FC = −1.5, FDR *p* < 0.05) from the WP DNA IRDAMAGE AND CELLULAR RESPONSE VIA ATR gene set and *MMP1* gene (RNA-seq FC = 2.4, FDR *p* < .05) from the STK33 UP gene set. Three of these genes are related to the cell cycle. The *TUBB* gene encodes β-tubulin protein, which is one of the major components of mitotic spindle (Parker et al., 2014). The *UBE2C* gene encodes one of the members of the anaphase-promoting complex, which is responsible for degradation of several target proteins along cell cycle progression (Nicolau-Neto et al., 2018). *CDK1* (cyclin-depended kinase 1) is one of the key parts of the cell cycle progression machinery (Kent and Leone, 2019), and it also participates in DNA repair (Johnson and Shapiro, 2010). *MMP1* is a matrix metalloproteinase that primarily breaks down native collagens and gelatin (Overall, 2002).

RT-PCR analysis of the organoids from Patient 1 confirmed the findings of the RNA-seq for all tested genes: *TUBB* (RT-PCR FC = −2.3, *p* < .05), *UBE2C* (RT-PCR FC = −1.3, *p* < .05), *CDK1* gene (RT-PCR FC = −2.3, *p* < .05), and *MMP1* gene (RT-PCR FC = 2.6, *p* < .05).

Interestingly, almost the same changes were observed for the HT-29 and RKO cell lines. The expression of the *MMP1* gene was too low in the cell lines to be measured with RT-PCR (the signal was lower than the threshold value after 40 cycles). However, the expression levels of the other genes were measured and demonstrated changes in the same direction. In HT-29 cells after the treatment with 9-ING-41, the expression of the *TUBB* gene decreased 2.6 times (*p* < .05), of the *UBE2C* gene 2.2 times (*p* < .05), and of the *CDK1* gene 1.8 times (*p* < .05). For the RKO cells, the changes were even more pronounced: *TUBB* (RT-PCR FC = −3.3, *p* < .05), *UBE2C* (RT-PCR FC = -2.1, *p* < .05), and *CDK1* gene (RT-PCR FC = −4.7, *p* < .05). Thus, our data demonstrate that the changes in mRNA profile after the treatment with 9-ING-41 are not specific for Patient 1 organoids and can be observed in other models of colorectal cancer.

## Discussion

Drug resistance is the principal factor that limits successful clinical outcomes in patients with cancer. Most patients with colorectal cancer receive chemotherapy based on cytotoxic antitumor effects. However, cancer is a multifactorial disease, and therefore, the treatment with just one class of drugs may not always be successful. Numerous studies of the changes in the regulatory mechanisms that control cell division in tumor cells reveal altered expression, aberrant cellular localization, and epigenetic dysregulations of previously known proteins and lead to reevaluation of their role in cancer. For example, the expression level of the antiaging gene Sirtuin 1 (Bosch-Presegué and Vaquero, 2014; Lee et al., 2019) was significantly associated with the depth of tumor invasion, differentiation, and tumor size in colorectal carcinoma (Yu et al., 2016; Sun et al., 2017; Simons et al., 2018). Currently, Sirtuin 1 is associated with cell stemness, DNA repair, telomere maintenance, and cell cycle control and is shown to be involved in transcriptional regulation of p53 by means of deacetylation (Mantel and Broxmeyer, 2008; Lee and Gu, 2013). Moreover, *Sirt1* is found to be coordinately overexpressed with KRAS and likely participates in the pathogenesis of endometriosis-associated ovarian cancer (Yoo et al., 2017; Teasley et al., 2020). Similarly to *Sirt1*, GSK-3β was also described to interact with p53, promote its actions, and be overexpressed in cancer cells expressing constitutively active KRas (Watcharasit et al., 2003; Zhang et al., 2011), which makes the GSK-3β/p53/Sirtuin 1 axis promising for future studies.

In the current work, we tested the ability of 9-ING-41, a small molecule inhibitor of GSK-3β, to decrease the growth rate of CRC cells with different KRAS-mutation status in a clinically relevant setting. Six CRC cell models were investigated: SW480 cell line with *KRAS* mutation (G12V), wild-type cell lines HT-29 and RKO, as well as three newly developed patient-derived CRC organoids. The morphological analysis of the latter confirmed the similarity of the initial tumor tissue and tumor organoids. DNA-seq data demonstrated complete conservation of the mutational profile between initial tumor tissue and tumor organoids with one exception for Patient 1. In this case a low content of cancer cells in the obtained tissue sample did not allow detecting somatic mutations. However, the only germline mutation was found in the tissue as well as in the corresponding tumor organoids. These data demonstrate that tumor organoids can be established and used for subsequent testing even in the case when content of cancer cells in the initial sample is very low. Moreover, two out of three established PD-TO lines contained common somatic mutations in *KRAS* gene: G12D (Patient 1) and G12C (Patient 3) (Jones et al., 2017). Interestingly, frequencies of the detected mutations were higher in tumor organoids in comparison with the original tissue. Moreover, the frequency of the most common mutation for each particular patient was almost 100% in organoids, and the frequencies of other mutations, adjusted to the most common one, were comparable between tissue and tumor organoids. It is well known that stromal cells do not survive in the culture conditions used for organoids (Drost and Clevers, 2018). Lack of Wnt ligands in the medium also favors growth of tumor cells rather than normal epithelial cells (van de Wetering et al., 2015). All these data suggest that only tumor cells survive *in vitro* and relative abundance of the clones harboring different mutations *in vitro* is almost the same as in the original metastatic tissue. In general, we show that morphological and genomic characteristics of the tumor are preserved well in tumor organoids consistently with previously published data (van de Wetering et al., 2015).

We did not observe any relationship between KRAS mutation status and sensitivity of the cell lines to 9-ING-41 alone and in combination with SoC drugs. The growth inhibitory effect of the combinations of 9-ING-41 with 5-FU and oxaliplatin on *KRAS* mutated (G12V) SW480 cells and *KRAS* wild-type HT-29 cells was comparable to SoC drugs. Notably, in the case of the *KRAS* wild-type RKO cells, which were more susceptible to SoC drugs and 9-ING-41 compared with SW480 and HT-29 cells, the addition of 9-ING-41 significantly improved the effect of 5-FU and oxaliplatin and their combination. In contrast, we have not found any significant benefit of 9-ING-41 and SoC drug combinations to the growth inhibition of the most sensitive organoids (Patient 2). However, organoids from Patient 2 were sensitive to a single treatment with 9-ING-41 or SoC drugs. Unlike cell lines, in the case of more resistant CRC organoids, 9-ING-41 significantly improved the results of the treatment. It was the only efficient drug for the organoids from Patient 1, and it also demonstrated an additive effect with 5-FU and oxaliplatin for the organoids from Patient 3. Overall, we have not found a pronounced specificity of 9-ING-41 toward *KRAS*-mutated cells that poses a challenge of finding molecular biomarkers of 9-ING-41 efficacy in CRC, but we do demonstrate that 9-ING-41 inhibits the growth of colorectal cancer cells via a distinct from chemotherapy mechanism of action.

These results are in good agreement with previously published data. It is shown that, in addition to its canonical function as a negative regulator of the Wnt/β-catenin pathway, GSK-3β can sustain proliferation and survival of CRC cells by poorly understood mechanisms (Shakoori et al., 2005). The expression of GSK-3β was elevated in colorectal tumor tissue in comparison to adjacent normal tissue almost in all patients both *KRAS* mutant and wild-type (Shakoori et al., 2005). Moreover, protein expression of GSK-3β was high in all tested CRC cell lines independent of their *KRAS* status (Shakoori et al., 2005). Inhibition of GSK-3β with other small molecule inhibitors (AR-A014418 and SB-216763) resulted in a decrease in viability of *KRAS* mutant CRC cell lines (SW480 and HCT116). In addition to a wide spectrum of cancer models, including glioblastoma (Ugolkov et al., 2017), pancreatic (Ding et al., 2017), breast (Ugolkov et al., 2016), and bladder (Kuroki et al., 2019) cancers, 9-ING-41 has already been tested on wild-type *KRAS* CRC cell line HT-29 (Poloznikov et al., 2019) and recently on a panel of CRC cell lines (Huntington et al., 2021). In both cases, it demonstrated an ability to decrease viability of colorectal cells. Our data confirmed these results in a more physiologically and clinically relevant setting on primary CRC organoids. Interestingly, now 9-ING-41 is being tested in a phase I/II clinical trial, including CRC patients (Safran et al., 2020). Despite possible suboptimal dosing regimen at the early stage of clinical trials, it is reported that one patient with CRC had stable disease after treatment with 9-ING-41 (Safran et al., 2020).

The results of the transcriptomic analysis support our findings of the growth inhibitory effect of 9-ING-41 and are in agreement with previously published studies. Changes in the profile of mRNAs clearly indicate inhibition of cell cycle resulting from action of 9-ING-41 (Figure 6; Table 3). According to the RT-PCR data, several selected genes related to the cell cycle were significantly downregulated in tumor organoids from Patient 1 as well as in colorectal cell lines after treatment with 9-ING-41. Specifically, the CDK-RB-E2F axis was suppressed. Previously, 9-ING-41 has been shown to arrest the cell cycle at G2/M in lymphoma (Wu et al., 2019) and bladder cancer cells (Kuroki et al., 2019). Moreover, similar to our results, expression of cyclin B1 and Cdk1, mitotic entry regulatory proteins, were downregulated in bladder cancer cell lines treated with 9-ING-41 (Kuroki et al., 2019). Our findings demonstrate that 9-ING-41 can arrest the cell cycle of CRC cells. Interestingly, we also found a lot of downregulated DNA repair–related gene sets in 9-ING-41 treated cells. Previously, it has been shown that 9-ING-41 prevents the activation of the ATR-DNA damage response pathway via activation of proteasome-dependent degradation of a critical ATR adaptor molecule TopBP1 (Ding et al., 2019). We also detected downregulation of this pathway in CRC organoids after treatment with 9-ING-41 (Figure 7; Table 4). Finally, there were several significantly downregulated gene sets related to the maintenance of telomeres (Figure 7; Table 4). This finding suggests that inhibition of GSK-3 by 9-ING-41 can prevent cancer cells from extension of telomeres, thus limiting their division (Guterres and Villanueva, 2020).

There are several hypotheses about the mechanisms how GSK-3β inhibition can enhance the effect of chemotherapeutic drugs. It is shown that GSK-3β inhibition leads to apoptosis *via* p53 activation in p53-wt HCT116 colon cancer cells (Tan et al., 2005) and a RT4 bladder cancer cell line (Kuroki et al., 2019). Although GSK-3β depletion had little effect on viability of p53-null HCT116p53KO colon cancer cells and p53-mut HT1376 bladder cancer cells, it restored the sensitivity of these cells to DNA-damaging agents, such as 5-FU. GSK-3β is described to be a positive regulator of NF-κB–mediated chemoresistance of cancer cells (Pecoraro et al., 2021). Therefore, mechanistically, inhibition or depletion of GSK-3β bypasses NF-κB–mediated drug resistance. Herein, we presume that GSK-3β inhibition leads to DNA repair disruption and plays a significant role in sensitizing of cancer cells toward 5-FU and Oxaliplatin.

Despite the fact that 9-ING-41 can be active on mutant as well as wild-type *KRAS* cancer cells, several reports suggest that GSK-3 can be specifically important for the malignant cells harboring mutations in the *KRAS* oncogene, and thus, inhibition of GSK-3 can be especially beneficial for the treatment of such cancers. Mutations in *KRAS* are the earliest events in pancreatic cancer initiation, and it is reported that expression of GSK-3b rises during progression of pancreatic cancer from preneoplastic lesions, suggesting a possible role for this oncogene in the observed overexpression (Ougolkov et al., 2006). Later, it was demonstrated that activation of the Ras–MAPK–ETS2–p300 cascade leads to GSK-3β overexpression in pancreatic cancer cells (Zhang et al., 2011). Moreover, it is shown that GSK3 is required for the *in vitro* and *in vivo* growth and survival of human mutant KRas-dependent tumors but may be dispensable for mutant KRas-independent tumors (Kazi et al., 2018).

Interestingly, another kinase named STK33 was also previously found to be critical for KRas-dependent cancer cells. Today, little is known about the role of STK33 in the biology of cancer. For the first time, the attention of cancer researchers on this kinase was attracted by the work of Scholl et al., in which the authors demonstrate dependence of KRAS mutant cancer cells on the expression of *STK33* (Scholl et al., 2009). It is shown that downregulation of *STK33* by shRNA leads to activation of apoptosis and a decrease in the growth rate of AML cells harboring mutant KRAS. In addition, knockdown of *STK33* in KRAS mutant epithelial cancer cell lines from different origins (including colorectal, breast, and pancreatic cancers) led to impaired colony formation *in vitro* and slower growth *in vivo*. Moreover, apoptosis in AML cells seemed to be mediated by S6K1 and dependent on kinase activity of STK33.

However, transient *STK33* knockdown by siRNAs turns out to have no effect on the viability of mutant KRAS-dependent cell lines (Babij et al., 2011). In addition, it is proved in several works that inhibition of kinase activity of STK33 by small molecule inhibitors does not actually suppress viability of KRAS mutant leukemic cells (Babij et al., 2011; Luo et al., 2012). On the other hand, it is demonstrated that inhibition of HSP90 (by shRNA or small molecules) led to a decrease in protein content of STK33 and reduction of viability *in vitro* and tumor growth rate *in vivo* for KRAS mutant CRC cell lines (Azoitei et al., 2012). All these data indicate that the nonkinase activities of STK33 can be responsible for its observed essentiality for the cancer cells harboring KRAS mutations. For example, it is shown that STK33 can bind to c-Myc and promote its activity (Yang et al., 2016).

In this work we show that inhibition of GSK3β with 9-ING-41 in *KRAS* mutant colorectal organoids leads to similar changes in the transcriptomic profile to the ones observed in *KRAS* mutant AML cells after suppression of *STK33*. Both these interventions were effective in prevention of the growth of malignant cells harboring *KRAS* mutations. This finding suggests a possibility that GSK3β might control the expression of STK33. We did not find any significant changes in mRNA level of *STK33* after treatment with 9-ING-41 (*p* = .42); however, protein content of STK33 could have changed. Another possibility is that both GSK3β and STK33 control the same downstream targets. One possible common downstream target is S6K as activity of this kinase is reduced after knockdown of STK33 in AML cells (Scholl et al., 2009) and in different cell types, including CRC cells HT-29, after knockdown or pharmacological inhibition of GSK3β (Shin et al., 2011). Further research will help to identify more detailed mechanism of interplay between these two kinases and their role in biology of *KRAS* mutant cancer cells.

## Conclusion

Our results suggest that 9-ING-41 could be an effective drug for the treatment of CRC. We demonstrate that 9-ING-41 inhibits the growth of CRC cells via a distinct from chemotherapy mechanism of action. Although molecular biomarkers of 9-ING-41 efficacy are yet to be identified, the addition of 9-ING-41 to the SoC drugs 5-FU and Oxaliplatin could significantly enhance growth inhibition in certain CRC cells. The results of the transcriptomic analysis support our findings of the growth inhibitory effect of 9-ING-41 in PD-TO and are in agreement with previously published studies. Transcriptomic analysis also revealed substantial similarities between suppression of STK33 and inhibition of GSK-3β. Both these kinases were previously identified as critically important for KRas-dependent cancer cells. Overall, the results of this study provide a rationale for the further investigation of GSK-3 inhibitors in combination with SoC treatment of CRC.

## Data Availability

The data sets presented in this study can be found in online repositories. The names of the repository/repositories and accession number(s) can be found below: https://www.ncbi.nlm.nih.gov/geo/, GSE184125.
